# Adsorption of Algal-Derived 2-Methylisoborneol (MIB) and Dimethyl Disulfide (DMDS) onto Activated Carbon: The Role of Pore Structure and Hydrophobicity

**DOI:** 10.3390/molecules30224348

**Published:** 2025-11-10

**Authors:** Yuqin Zhao, Yulan Zhao, Hui Guo, Denghui Peng, Wenwen Kong, Fengjian Yan, Shumei Zhou, Quansheng Li, Boxiong Shen, Chongrui Lyu

**Affiliations:** 1 Tianjin Water Group Limited Company, Yinluan Erwangzhuang Branch, Tianjin 301802, China; 2 Hebei Engineering Research Center of Pollution Control in Power System, School of Energy and Environmental Engineering, Hebei University of Technology, Tianjin 300401, China; 3 Tianjin Key Laboratory of Clean Energy and Pollution Control, School of Energy and Environmental Engineering, Hebei University of Technology, Tianjin 300401, China

**Keywords:** activated carbon, 2-methylisoborneol (MIB), dimethyl disulfide (DMDS), Pore-filling, hydrophobicity, water treatment, Odor control

## Abstract

2-methylisoborneol (MIB, d = 0.6 nm) and dimethyl disulfide (DMDS, d = 0.7 nm) produced by algal metabolism are the main olfactory contaminants of drinking water. Activated carbon (AC) adsorption is an effective method to remove MIB/DMDS, yet critical gaps remain regarding the dominant factors and mechanisms governing their different adsorption performance. The microporous filling mechanism is the dominant mechanism for the adsorption of MIB and DMDS by AC. Surface functional groups play a supporting role in the adsorption process by modulating the hydrophilicity/hydrophobicity of the carbon surface. This study systematically evaluated the adsorption performance of three ACs—coconut shell-derived (CSC), coal-based (CAC), and Sargassum-derived (SAC)—for MIB and DMDS removal. Comparative analysis revealed the superior adsorption performance of CSC, achieving 87.41% removal of MIB and 71.2% removal of DMDS at 20 mg/L. Both MIB and DMDS adsorption adhere to the Langmuir isotherm, indicating monolayer coverage with uniform energy. Kinetic studies demonstrated that the PSO model fits the MIB adsorption process best, while the PFO model fits the DMDS adsorption process best. The FTIR confirmed physical adsorption, with no new chemical bonds formed. Furthermore, regenerated CSC retains significant adsorption capacities, achieving 85.89% and 68.49% of the original capacity for MIB and DMDS, respectively, after five regeneration cycles. This research provides fundamental insights into the mechanistic role of AC properties in odorant removal processes, supporting its sustainable application in water treatment.

## 1. Introduction

The accelerated development of industrialization and urbanization has exacerbated aquatic eutrophication and odor pollution, thereby severely compromising ecological integrity and adversely affecting the quality of life for residents. These malodorous phenomena primarily result from secondary metabolites generated by excessive growth of algae and bacteria in aquatic systems [1]. Among typical taste and odor compounds (T&O), 2-methylisoborneol (MIB) and dimethyl disulfide (DMDS) have received widespread attention due to their unique hydrophobicity and ultra-low odor thresholds (10 ng/L and 0.1 μg/L, respectively) [2,3]. MIB imparts a persistent earthy–musty odor, whereas DMDS produces offensive sulfurous notes. Both compounds degrade drinking water aesthetics at trace concentrations and may pose human health risks via bioaccumulation pathways [4].

Activated carbon (AC) adsorption exhibits superior performance in removing most contaminants, including T&O such as MIB and DMDS [5,6,7]. While coal-based activated carbon (CAC) is mostly used to absorb T&O, and CAC can adsorb up to 22 ng/mg of MIB [8], the comparative performance of alternative AC sources—particularly sustainable and cost-effective materials—remains underexplored [5,6,7]. Coconut shell activated carbon (CSC), for instance, offers advantages such as high surface area, mechanical strength, and abundant availability, making it a promising candidate for large-scale water treatment applications. Also, the ecological issues caused by cyanobacterial blooms and the underutilization of algal biomass necessitate innovative approaches to resource recovery and sustainable adsorbent development. To address the gaps, this study investigates the adsorption mechanisms of MIB and DMDS on AC while also introducing a novel porous AC derived from Sargassum (SAC) as an eco-friendly alternative.

The adsorption mechanisms of algae-derived odorous substances on AC remain debated. For example, in terms of the adsorption of MIB by ACs, Matsui et al. and Yu et al. demonstrated that the pore-filling mechanism governs the adsorption progress [8,9]. Huang et al. [10] proposed that nitrogen-doped AC exhibited high MIB affinity due to π-π electron donor–acceptor (EDA) interactions. However, Yu et al. [8] reported that there was no correlation between the MIB adsorption capacity of ACs and AC’s oxygen content or functional groups. For DMDS, most research has focused on environmental applications (e.g., odor control or catalysis), with limited research on its adsorption by AC. Cui et al. [11] emphasized the role of acidic oxygenated functional groups for the adsorption of DMDS, whereas Huang et al. [5] argued that π-π EDA interactions with the AC’s graphene structure may dominate the adsorption of DMDS, downplaying the importance of functional groups.

According to the above background, existing research remains controversial regarding the adsorption mechanisms of MIB and DMDS on activated carbon, and systematic comparisons of the performance of activated carbon from different sources are lacking. Therefore, this study aims to determine the most promising adsorbent and then thoroughly investigate their adsorption mechanisms, with a particular focus on the synergistic effects of pore structure and surface properties. This study conducted a comprehensive investigation of three ACs (CSC, CAC, and SAC) for aqueous-phase removal of MIB and DMDS. The physicochemical properties of the materials were elucidated by observing the surface morphology characteristics (SEM), determining their specific surface area and pore volume (N_2_ adsorption–desorption analysis), crystal structure (XRD), and the type and content of surface functional groups (FTIR and Boehm titration). The effects of AC dosage, contact time, solution pH, and temperature on MIB and DMDS adsorption by AC were discussed, while kinetic analyses and isothermal modeling were conducted. The study aims to reveal mechanisms underlying MIB and DMDS adsorption. This work provides fundamental insights into the role of AC properties in odorant removal processes.

## 2. Results and Discussion

### 2.1. Characterization Results

#### 2.1.1. Microstructural Analysis

**Figure 1:**
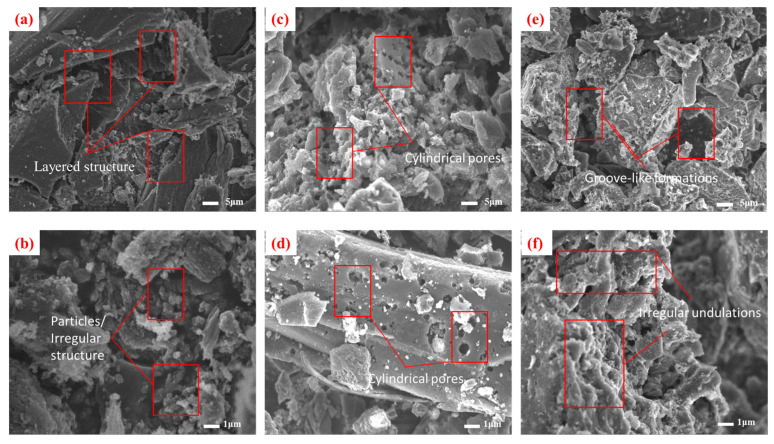
SEM images of different carbon materials: (**a**,**b**) CSC; (**c**,**d**) CAC; (**e**,**f**) SAC.

Microstructural analysis revealed distinct morphological characteristics among the three ACs. CSC displays a well-defined lamellar architecture, with compactly stacked graphitic layers (Figure 1a,b). This structural organization facilitates substantial specific surface area development, complemented by irregular protrusions and angular surface features [12].

CAC exhibits a hierarchical porous network featuring cylindrical, wedge-shaped, and bottle-shaped pores with nanoscale diameters [13] (Figure 1c,d). These pores are interconnected through microchannels, providing channels for the fixation of MIB/DMDS on the micropores, thereby improving mass transfer efficiency, though localized particle aggregation creates microscale clusters.

SAC presents a markedly heterogeneous surface morphology, characterized by irregular undulations and groove-like formations (Figure 1e,f). Its fragmented microstructure contains both mesoporous regions and structural collapse features, with particle dimensions spanning sub-micron to several micrometers. The surface displays numerous nanoscale fissures and defect sites, while the overall porosity appears less organized compared to CSC and CAC [14]. These morphological variations significantly influence the materials’ adsorption behaviors through differential pore accessibility.

#### 2.1.2. Specific Surface Area and Pore Volume

**Figure 2:**
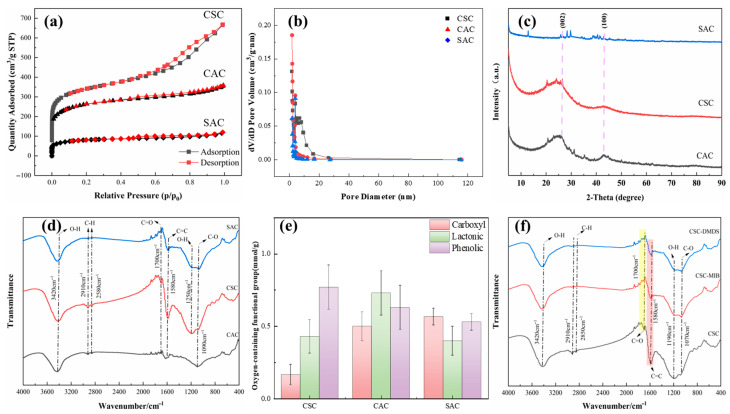
(**a**) Adsorption isotherm of N_2_. (**b**) Pore size distribution calculated by the NLDFT method. Square: CSC; triangle: CAC; diamond: SAC. (**c**) XRD of different types of AC. (**d**) FTIR analysis of different types of AC. (**e**) Boehm titration results for different types of AC. (**f**) Effect of single-solute adsorption experiments on functional groups on the surface of AC.

The pore structure of AC is categorized into macropores (pore diameter (d) > 50 nm), mesopores (2 < d < 50 nm), and small pores (d < 2 nm) [15]. Figure 2a presents the N_2_ adsorption–desorption isotherms for the three AC variants, revealing distinct adsorption capacities in the order CSC > CAC > SAC. The nitrogen adsorption isotherm of CSC exhibits a Type IV isotherm, which is a typical characteristic of mesoporous materials. CAC and SAC exhibited Type I behavior, proving that they are microporous materials [16].

The pore size distribution obtained via BJH analysis of the desorption curve (2–270 nm) is shown in Figure 2b. Although CSC contains a substantial amount of mesoporous structure, its micropore volume still amounts to 0.363 cm^3^/g. The overall pore volume of CAC is low, and the pore size is concentrated in a small range (1–3 nm), indicating that the proportion of CAC micropores (<2 nm) is high, but the pore structure is relatively single and the total volume is limited. The pore size distribution of SAC and CAC overlaps but is more dispersed, indicating that SAC is mainly microporous, and the pore structure is similar to CAC. Notably, CSC displayed higher macropore abundance compared to CAC and SAC (Table 1). While macropores facilitate molecular diffusion, they contribute minimally to adsorption capacity [17].

Multi-point BET analysis further quantified structural parameters: CSC exhibited the largest total specific surface area (SSA), micropore surface area (Smicro), and total pore volume (Vt) (CSC > CAC > SAC) (as shown in Table 1). CSC and CAC exhibit similar pore volume and pore volume, while SAC is much smaller than CSC and CAC in both total pore volume and pore volume. Previous studies have shown that the micropores of PAC will significantly affect its adsorption capacity for MIB/DMDS, so micropores may be an important parameter affecting the adsorption capacity [5].

#### 2.1.3. Crystal Structure

XRD analysis (Figure 2c) identified characteristic crystalline phases in the AC. All ACs exhibited broad peaks at approximately 26° and 43°, corresponding to the (002) and (100) crystallographic planes of graphitic carbon, confirming their predominantly amorphous nature [2]. SAC displayed unique diffraction signatures: a weak peak at 13° indicative of disordered carbon stacking or residual graphitic oxide structures, and a minor peak near 32° likely originating from silicon impurities inherent to its algal precursor [18].

Low-angle XRD intensity variations further corroborated porosity characteristics observed in nitrogen physisorption (BET). The pronounced low-angle scattering intensities for CSC and CAC reflect their well-developed microporous architectures, consistent with BET surface area data [19] (Table 1). In contrast, SAC displayed attenuated low-angle signal aligns with its reduced microporosity and a more heterogeneous pore distribution.

#### 2.1.4. Surface Functional Group Type and Content

The surface chemistry of the AC was qualitatively measured by FTIR as shown in Figure 2d. The peak at 3420 cm^−1^ represents the O-H bond stretching vibration [20]. The absorption peaks at 2910 cm^−1^ and 2580 cm^−1^ originate from the C-H asymmetric and symmetric stretching vibration, which are usually not directly involved in the reaction. The peak of CSC at 1700 cm^−1^ has a more pronounced peak attributed to the C=O stretching vibration [21]. In comparison with CAC and SAC, CSC evinces a significant enhancement in the vibration of C=C at 1580 cm^−1^ [22], originating from the conjugated structure of the AC (e.g., the vibration of graphene domains). The stretching vibration at 1250 cm^−1^ then originates from the OH within the -COOH. The peak at 1090 cm^−1^ may be attributed to the stretching vibration of different C-O bonds. Huang et al. proposed that the absence of the C≡C functional groups might result in improved DMDS adsorption [5]. However, the present study did not detect characteristic C≡C vibrations near 2347 cm^−1^, suggesting further studies are required to confirm this observation.

The results of the Boehm titration experiment are shown in Figure 2e. Oxygen-containing functional groups modulate the adsorption performance of ACs through dual mechanisms: (1) direct chemisorption interactions with target contaminants (e.g., via covalent bonding or ion exchange), and (2) indirect regulation of surface hydrophobicity, which governs the partitioning behavior of nonpolar adsorbates (e.g., log *K_ow_* > 3.0) at the solid–liquid interface. An increase in oxygen-containing functional groups correlated with reduced hydrophobicity, a characteristic detrimental to the partitioning behavior of hydrophobic compounds such as MIB and DMDS [23].

The lowest concentration of functional groups was presented by CSC, with a total oxygenated functional group content of 1.37 mmol/g, containing only 0.17 mmol/g of carboxyl groups, and a phenolic group concentration of 0.77 mmol/g. The contents of carboxyl groups in CAC and SAC were found to be 0.50 and 0.57 mmol/g, respectively, with phenolic group concentrations measuring 0.63 and 0.53 mmol/g. The CSC total oxygenated functional group content is lower than that of the other two. This suggests that the surface of the material is the most hydrophobic and is less likely to be covered by aqueous films. Furthermore, its abundant micropores are more effectively exposed to hydrophobic 2-MIB and DMDS molecules.

### 2.2. Influencing Factors

#### 2.2.1. Adsorption Capacity Optimization

**Figure 3:**
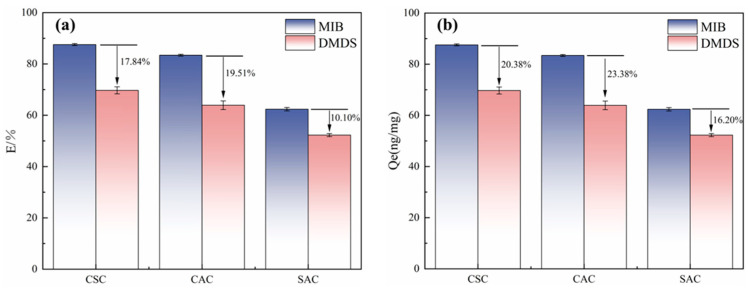
(**a**) Adsorption efficiency of different types of AC on MIB and DMDS. (**b**) Unit adsorption capacity of different types of activated carbon for MIB and DMDS.

The adsorption capacities of MIB and DMDS by different ACs at 20 mg/L dosage were calculated as shown in Figure 3. It can be seen that the adsorption capacity of MIB is stronger than that of DMDS across all tested ACs, regardless of their properties. This phenomenon is identical to the experimental results of Huang et al. [5]. The reason for this phenomenon may be the difference in molecular structure and hydrophobicity between the two. The octanol/water partition coefficients (*K_ow_*) of MIB and DMDS were 3.31 and 1.77, respectively, which indicates that MIB is more hydrophobic and more easily adsorbed by ACs [5]. Previous studies have suggested that the acidic functional groups will inhibit MIB adsorption by making AC polarized to attract water molecules [5,24].

Comparative analysis of adsorption capacities (Figure 3) demonstrated CSC’s superior performance for both MIB and DMDS removal relative to CAC and SAC. This performance level directly correlates with CSC’s structural advantages identified through BET characterization—specifically, its hierarchical pore architecture combining substantial microporosity with optimized meso/macropore networks (Table 1). While providing abundant adsorption sites, CSC is a superior adsorption material compared to CAC and SAC [25]. Previous studies have suggested that the acidic functional groups will inhibit MIB adsorption by making AC polarized to attract water molecules [5,24].

#### 2.2.2. Dosage

**Figure 4:**
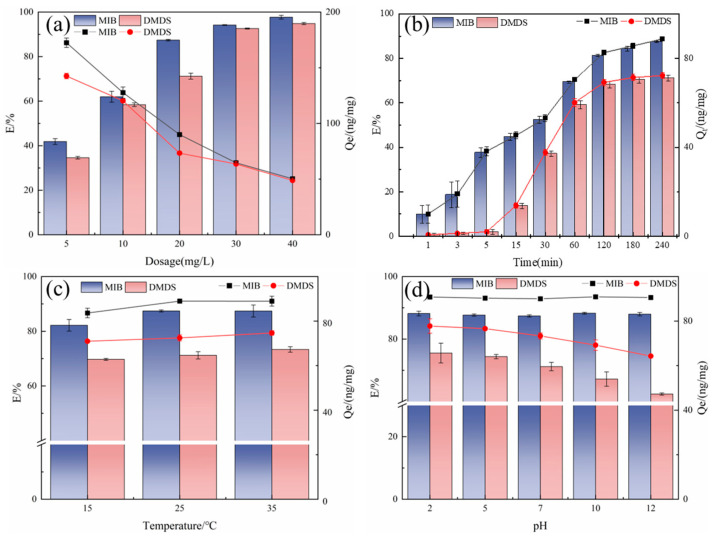
Effect of different factors on the adsorption of MIB and DMDS by CSC (**a**) dosage; (**b**) adsorption time; (**c**) temperature; (**d**) pH.

The effect of adsorbent dosage on the removal of MIB and DMDS is presented in Figure 4a. As expected, the removal efficiency for both compounds increased with increasing CSC dosage, from approximately 35–40% at 5 mg/L to over 95% at 40 mg/L. This trend is attributed to the greater availability of adsorption sites and surface area at higher dosages.

However, the unit adsorption capacity (*Q_e_*) exhibited a contrasting trend, decreasing significantly as the dosage increased. This phenomenon is characteristic of adsorption systems approaching site saturation. At lower dosages, the fixed amount of pollutant molecules is efficiently partitioned onto a limited number of high-affinity sites, resulting in a high *Q_e_*. At higher dosages, while the total number of sites increases, the pollutant-to-adsorbent ratio decreases, leading to a fraction of sites remaining underutilized and thus a lower average capacity per unit mass of adsorbent [26,27].

Notably, MIB consistently demonstrated a higher removal efficiency and unit capacity than DMDS across all tested dosages. This observation aligns with the higher hydrophobicity of MIB (*K_ow_* = 3.31) compared to DMDS (*K_ow_* = 1.77), as discussed in Section 2.2.1, which favors its partitioning from the aqueous phase onto the hydrophobic carbon surface [5].

#### 2.2.3. Adsorption Time

Longer contact times increase adsorption efficiency and unit adsorption capacity, as this allows more active sites on the adsorbent surface to be utilized (Figure 4b). The adsorption rate of MIB was significantly higher than that of DMDS at all adsorption times, indicating that MIB is more suitable for adsorption by ACs [5].

During the initial rapid uptake phase (0–60 min), MIB and DMDS removal rates reached 69.33% and 59.21%, respectively, driven by abundantly available adsorption sites and favorable concentration gradients enabling efficient micropore filling [28]. During the rapid adsorption phase, the adsorption of MIB has been in a trend of steady growth, but DMDS was hardly adsorbed in the first 5 min, and a significant adsorption behavior was observed only after 15 min. It may be due to the low hydrophobicity of DMDS, the initial adsorption drive is insufficient, and it takes longer to penetrate through the aqueous film on the surface of the AC and enter the pore space.

Subsequently, adsorption transitioned to an equilibration phase (120–240 min) as surface sites became progressively occupied. As the adsorption sites on the surface of the adsorbent were gradually occupied by MIB/DMDS, the diffusion rate of MIB/DMDS decreased, and the adsorption rate of the AC gradually decreased. Equilibrium was achieved at 240 min, marking the establishment of dynamic equilibrium between adsorption and desorption processes.

#### 2.2.4. Temperature

Temperature-dependent adsorption behavior was evaluated across environmentally relevant conditions (15–35 °C). As shown in Figure 4c, the adsorption efficiency increases with rising temperature: MIB removal rate increased from 82.17% (15 °C) to 87.39% (35 °C), while DMDS removal rate rose from 69.71% to 73.35% over the same range. This indicates that AC is heat-absorbing during the adsorption of MIB/DMDS, and the adsorption capacity increases with increasing temperature [29]. Despite this thermal enhancement, there is a limited magnitude of efficiency gains (≤8% total variation across the 20 °C range) [30]. This result was supported by Yuan et al. [31] and Wu et al. [32]. Based on these findings, subsequent experiments standardized conditions at 25 °C, a representative median temperature balancing adsorption efficiency with operational feasibility.

#### 2.2.5. pH

The pH usually affects the adsorption process by changing the charge state of the AC surface and the ionic form of the adsorbent [33]. As illustrated in Figure 4d, the removal of MIB remained stable across pH 2–12 (78.2–82.4%). The study by Li et al. [34] also showed that the effect of pH on MIB adsorption was very limited.

In contrast, DMDS adsorption exhibited significant pH dependence. The highest DMDS removal efficiency (79.18%) was observed under strongly acidic conditions (pH 2), declining progressively to 62.43% at pH 12. This difference in adsorption behavior at elevated pH is mainly due to the hydrophobicity of the two compounds [11]. pH change mainly affects the hydrophilicity/hydrophobicity of the AC surface, and despite the increased hydrophilicity of the AC surface at high pH, the strong hydrophobicity of the MIB can overcome the resistance of the hydration layer on the surface, whereas the weak hydrophobicity of the DMDS results in a more sensitive response to the pH change [35].

### 2.3. Adsorption Process and Kinetic Analysis of Three Carbon Materials

#### 2.3.1. Adsorption Kinetics

**Figure 5:**
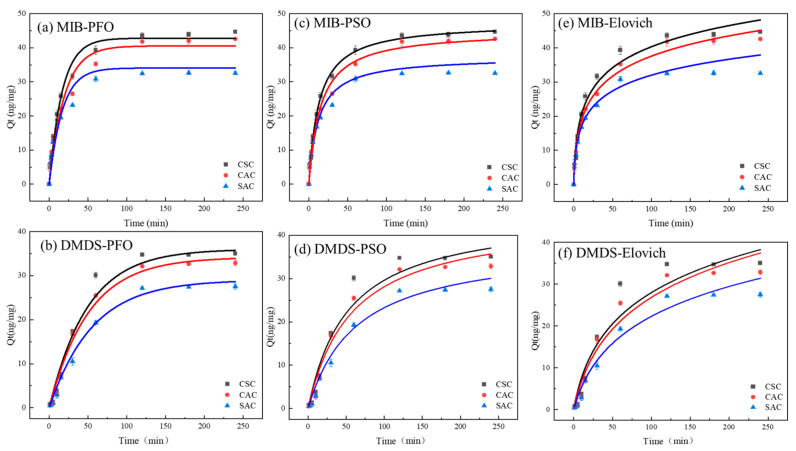
(**a**,**b**) PFO model; (**c**,**d**) PSO model; (**e**,**f**) Elovich model.

The PFO model, PSO model, and Elovich model were used to fit the experimental results, respectively, and the fitting curves are shown in Figure 5, and the corresponding data are shown in Table 2. The PFO model is an empirical model that simulates the ideal fast physical adsorption process for a monolayer [36]. Notably, the PFO model demonstrated strong correlations, with *R*^2^ values exceeding 0.94 for MIB and 0.99 for DMDS.

The PSO model is an empirical model based on adsorption capacity. It assumes that the adsorption rate is determined by the square of the number of unoccupied active sites on the adsorbent surface and is typically associated with chemical adsorption processes [37]. The PSO model also exhibited high precision, with *R*^2^ values greater than 0.97 for MIB and 0.98 for DMDS. In the case of CSC, the PSO model is more suitable for describing the adsorption of MIBs on CSCs, whereas DMDS is more suitable for PFO modeling (Table 2).

The Elovich model is an empirical model that takes into account a series of reactions, such as activation or diffusion of a solute at an interface or in a solution, as well as surface deactivation [38]. In this experiment, the Elovich model fitted the adsorption data of the three ACs on MIB and DMDS very well, and the *R*^2^ curves fitted to MIB and DMDS were all greater than 0.97. This indicates that there is an involvement of activation energy in the adsorption process of the two olfactory substances, and the process of adsorption is a very complex chemical reaction that is likely to occur during the adsorption process [39].

**Figure 6:**
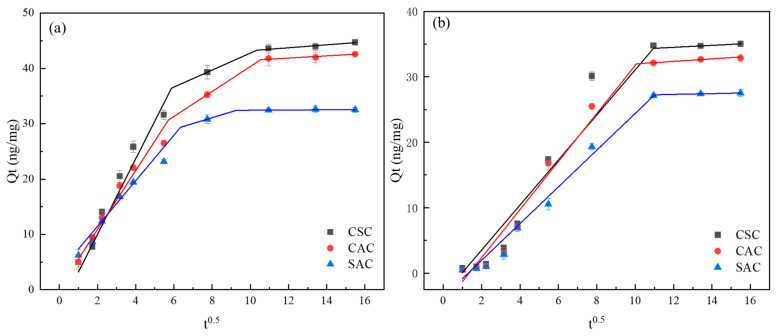
Weber–Morris model: (**a**) MIB; (**b**) DMDS.

While the aforementioned kinetic models describe the overall adsorption rate, they do not explicitly identify the diffusion mechanism. To gain further insight into the rate-controlling steps and the diffusion mechanism involved in the adsorption process, the kinetic data were further analyzed using the Weber–Morris intra-particle diffusion model. As shown in Figure 6, the Weber–Morris model was modified with *t*^0.5^ as the horizontal coordinate and subjected to time-segmented linear fitting. Three adsorption phases were observed for MIB (Figure 6a), while two adsorption phases were observed for DMDS (Figure 6b). Figure 6 reveals that the first adsorption stage exhibits the steepest slope, with the slope of the second or third stage gradually decreasing over time (Table 2). This sequential variation in adsorption rate can be attributed to the greater mass transfer dynamics during the initial adsorption phase and the availability of multiple adsorption sites on the adsorbent. During the first stage, adsorbate molecules diffuse through the liquid film to the outer surface of the adsorbent particles. When the curve passes through the origin, intra-particle diffusion can be considered the sole rate-limiting step. As shown in Figure 6, the curves for MIB and DMDS do not pass through the origin, indicating that intra-particle diffusion is an important rate-limiting step but not the sole controlling factor, with membrane diffusion also contributing to the process control. In the second stage, the adsorbate diffuses within the pores of the particle. The third stage corresponds to the adsorption equilibrium phase, where nearly all adsorption sites within the particle are occupied, diffusion rates become extremely slow, and dynamic equilibrium is ultimately reached. The findings from the Weber–Morris model corroborate the conclusions from the PFO and Elovich models, indicating that the adsorption process is complex and involves multiple stages, with both film diffusion and intra-particle diffusion playing significant roles. This multi-stage diffusion behavior is consistent with the physical adsorption of MIB and DMDS within the hierarchical pore structure of the ACs.

#### 2.3.2. Adsorption Isotherms

The Langmuir model is a theoretical adsorption formula based on the assumption that adsorption occurs on a uniform surface [40]. As shown in Table 3, the Langmuir model for all three ACs can fit the adsorption of MIB and DMDS on ACs well, so it can be assumed that there is a monolayer adsorption of ACs on these two olfactory substances [41]. The equilibrium parameter, *R_L_*, was used to determine the adsorption capacity of AC for the two olfactory odors; when *R_L_* was equal to 0, the adsorption was irreversible, and when 0 < *R_L_* < 1, the adsorption was effective. As shown in Table 3, the *R_L_* of the three ACs for the two odorants was much less than 1 [42]. Therefore, it can be further verified that the ACs used in this experiment can effectively adsorb the two odorants.

**Figure 7:**
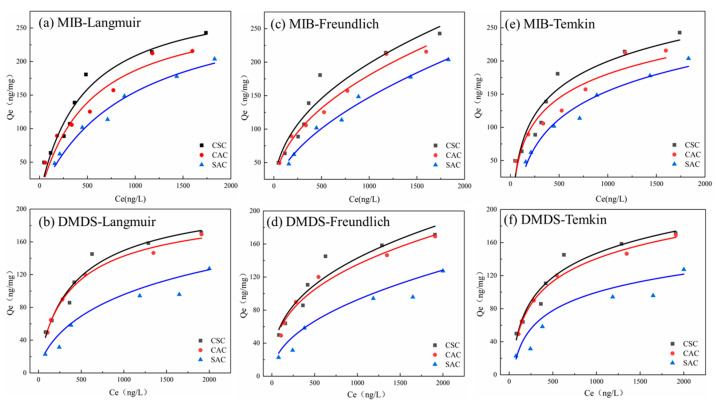
(**a**,**b**) Langmuir model; (**c**,**d**) Freundlich model; (**e**,**f**) Temkin model.

The Freundlich model is an empirical model, which is especially suitable for the adsorption of hydrophobic adsorbents [43]. As shown in Figure 7b and Table 3, the Freundlich model is in good agreement with the present experimental data, and its *R*^2^ is greater than 0.9. Therefore, it can be concluded that the adsorption of MIB/DMDS onto AC involves multilayer adsorption [44]. The parameter 1/n can show the size of adsorption strength; the smaller 1/n is, the stronger the force between adsorbent and adsorbate is [44]. As shown in Table 3, 0.1 < 1/n < 1, indicating that surface adsorption reactions occur easily [45].

The Temkin isotherm model is a two-parameter thermal theory model [46], and the fitted curves *R*^2^ of both MIB and DMDS were greater than 0.91; the fitted curves could fit the experimental data well, which indicated that the adsorption of these two olfactory substances by AC was accompanied by the change in energy. The binding energies (*K_T_*) and isothermal constants of MIB and DMDS are shown in Table 3. In the case of physical adsorption, the adsorbate attaches to the adsorbent solely through weak van der Waals forces. Consequently, the adsorption energy in physical adsorption processes is relatively low (below 25 kJ mol^−1^) [46]. The low *B_T_* values observed in this study are consistent with findings from other models (kinetic and thermodynamic), further supporting the conclusion that the adsorption of MIB and DMDS onto AC primarily involves physical adsorption processes.

As seen above, all three isothermal adsorption models exhibited some explanatory power for the adsorption of MIB and DMDS on AC, revealing the complexity of the adsorption mechanism. The good fit of the Langmuir model (*R*^2^ > 0.9) suggests that adsorbates may form a monomolecular layer covering the surface of the AC, hinting at the existence of uniform surface adsorption sites [41]. The high fit of the Freundlich model (*R*^2^ > 0.9) reveals the multilayer adsorption characteristic of the adsorption process, especially the parameter 1/n between 0.1 and 1, which not only suggests the strong force between adsorbent and adsorbate but also reflects the existence of energetic heterogeneity on the AC surface, which may originate from the non-uniformity of its porous structure [44]. This seemingly contradictory model coexistence phenomenon suggests that the actual adsorption process may simultaneously have the synergistic effect of monolayer and multilayer adsorption, or different regions of the surface show differentiated adsorption behaviors.

#### 2.3.3. Adsorption Thermodynamics

The adsorption isotherms and parameters of CSC at different temperatures are shown in Supplementary Material Figure S3 and Table S4. As shown in Table S4, the negative values of ΔG across all tested temperatures (288 K to 308 K) confirm that the adsorption process is spontaneous and thermodynamically favorable. The magnitude of ΔG became more negative with increasing temperature, indicating that the spontaneity of the adsorption process was enhanced at higher temperatures. which also explains the phenomenon that the maximum adsorption amount of AC on these two olfactory odors is gradually increased with the increase in temperature [47]. This enhanced spontaneity can be understood by considering the other two thermodynamic parameters. The positive value of ΔH indicates that the adsorption process is endothermic. Simultaneously, the positive value of ΔS suggests an increase in randomness at the solid–liquid interface during adsorption, likely due to the release of previously organized water molecules from the hydrophobic carbon surface and the hydration shells of the MIB/DMDS molecules (hydrophobic effect).

The thermodynamic calculation results are shown in Table 4. The calculation process is shown in Figure S4 and Table S5. The enthalpy changes (ΔH) of CSC for MIB and DMDS adsorption are 8.4928 kJ/mol and 8.8361 kJ/mol, respectively. Considering that physical adsorption processes typically exhibit ΔH values below 25 kJ/mol [48], combined with the preceding kinetic and isotherm analyses, it can be concluded that the adsorption mechanism of CSC toward MIB/DMDS is primarily governed by physical adsorption.

### 2.4. Mechanism Analysis

**Figure 8:**
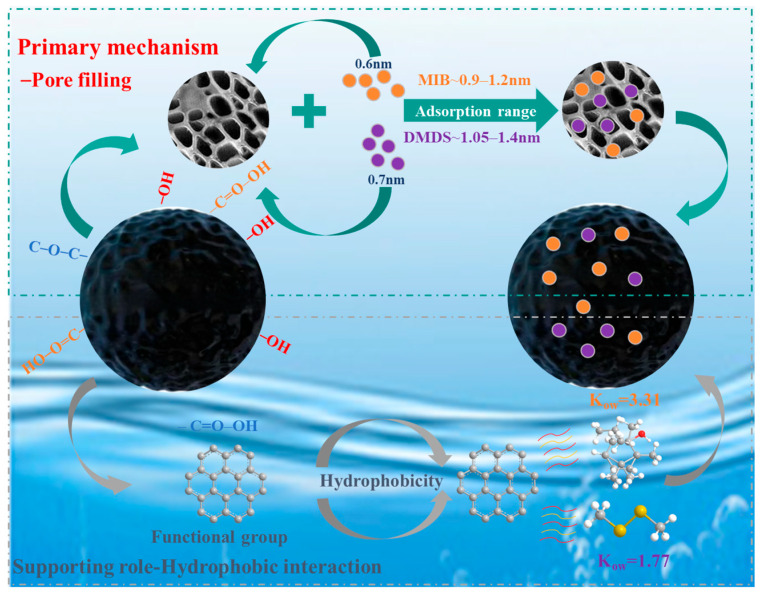
Adsorption mechanism of MIB and DMDS on AC.

The microporous filling mechanism is the dominant mechanism for the adsorption of MIB and DMDS by AC, and the adsorption capacity mainly depends on the specific surface area and pore volume of the micropores of the AC. Although CSC (V_micro_ = 0.3630 cm^3^/g) and CAC (V_micro_ = 0.3710 cm^3^/g) share similar microporous structures, CSC exhibits superior adsorption performance for MIB and DMDS compared to CAC. This enhancement is attributed to its more developed mesoporous structure, which provides efficient pathways for the adsorption of MIB/DMDS (Table 1). In contrast, the microporous structure of SAC (V_micro_ = 0.1086 cm^3^/g) is underdeveloped, resulting in lower adsorption capacity than both CSC and CAC. This strongly suggests that micropores serve as the primary adsorption sites, which is in agreement with the molecular size–pore matching relationship [49]. As shown in Figure 8, the molecular diameters of MIB and DMDS are approximately 0.6 nm and 0.7 nm, respectively. Based on the relationship [49], the optimal pore size for adsorption is 1.5–2 times the molecular diameter, corresponding to 0.9–1.2 nm for MIB and 1.05–1.4 nm for DMDS (Figure 8). These intervals lie entirely within the microporous region (<2 nm).

Also, surface functional groups play a supporting role in the adsorption process by modulating the hydrophilicity/hydrophobicity of the carbon surface. The adsorption capacities of both MIB and DMDS decreased with increasing carboxyl group concentration on the AC. In Park’s study, oxygen-containing functional groups impart hydrophilic character to the AC surface, facilitating the formation of water clusters via hydrogen bonding in aqueous environments, leading to pore blockage and reduced accessibility for hydrophobic adsorbates [50]. However, in the present study, no significant correlation was found between the adsorption capacity and the concentration of oxygen-containing functional groups. This lack of correlation suggests that pore filling likely served as the dominant mechanism. FTIR spectroscopy performed on the CSC sample—as the representative and best-performing adsorbent—provided additional evidence: no new chemical bonds (e.g., C–S or S–H) were detected after MIB/DMDS adsorption (Figure 2f). This supports the inference that physical adsorption is the dominant mechanism for CSC, a conclusion that is consistent with the kinetic, isotherm, and thermodynamic data obtained for all three ACs. While it strongly suggests the absence of chemisorption for this material, the adsorption mechanisms on the other adsorbents warrant further spectroscopic characterization in future studies.

### 2.5. Regeneration

**Figure 9:**
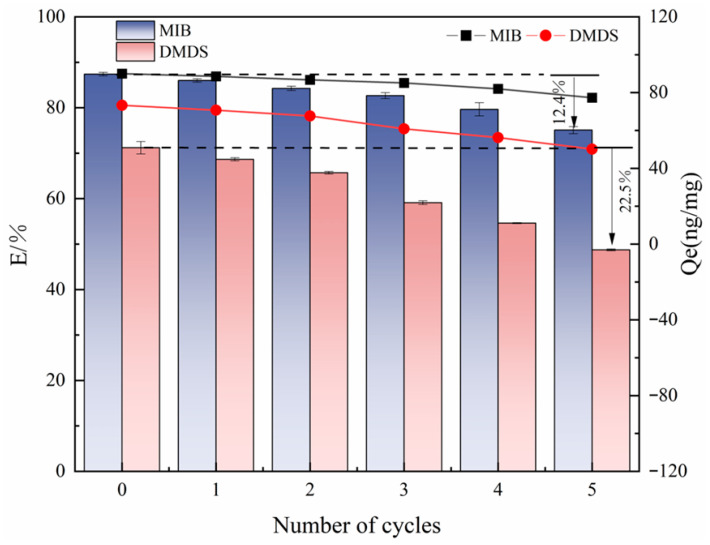
Effect of the number of cycle regeneration on the adsorption of MIB and DMDS by CSC.

The reusability evaluation in this study was focused on the best-performing adsorbent (CSC). To this end, its cyclic regeneration performance was evaluated by a five-cycle adsorption–desorption experimental system. The quantitative analysis results showed (as shown in Figure 9) that the *Q_e_* of CSC on MIB only decreased by 5.4% after three cycles. A more pronounced decreasing trend was observed after the fourth cycle, while the adsorption of DMDS by CSC could only be maintained at a high level in the first two cycles. After five cycles, the adsorption capacity retention of CSC for MIB remained at the level of >75% after the complete cycle test, which was only decreased by 12.4% compared with the initial value. *Q_e_* decreased by 12.69 ng/mg, demonstrating good cyclic stability. However, the adsorption efficiency for DMDS showed a significant decay trend from the initial 71.20% to 48.74%. *Q_e_* decreased by 23.13 ng/mg. This differential cycling performance may be related to the limited solubility of DMDS in methanol solvent—its lower solubility characteristics led to restricted desorption kinetics, resulting in incomplete regeneration of the active site. Future work should include a comparative analysis of the regeneration potential of different activated carbons to comprehensively evaluate their economic and practical feasibility.

## 3. Experimental Materials and Methods

### 3.1. Experimental Materials

CSC and CAC were purchased from Tianjin Purite Purification Technology Co., Ltd (Tianjin, China). *Sargassum* biomass was purchased from the Shanghai Guangyu Biotechnology Co., Ltd. (Shanghai, China). and washed with deionized water to remove impurities, dried at 80 °C for 24 h, and pyrolyzed at 650 °C for 2 h under N_2_ atmosphere. The prepared carbon was activated by mixing with KOH (mass ratio 1:3) under continuous heating and stirring for 1 h and washed with 2 M of HCl. The AC derived from *Sargassum* (SAC) were milled and sieved to 200 mesh, washed with deionized water until the pH reached 7, and then dried at 105 °C for 24 h.

MIB and DMDS were purchased from Anpu Cloud Laboratory Products (Shanghai) Co., Ltd., China, at a concentration of 100 mg/L in methanol. Experimental solutions of 2000 ng/L were prepared by diluting the methanol solution with ultra-pure water (resistivity ≥ 18 mΩ cm). The basic physical and chemical properties are shown in Table S1.

### 3.2. Adsorption Experiment

Experimental solutions of MIB and DMDS (2000 ng/L) were prepared using pure water. For each adsorption experiment, 500 mL of the stock solution was placed in a 500 mL conical flask. The solution was stirred thoroughly and allowed to stand for 10 min to stabilize. The initial concentration (*C*_0_) for all adsorption calculations was defined as the concentration of MIB and DMDS measured in the 0 mg/L control experiment after the complete adsorption period (240 min). The effects of key parameters—including activated carbon (AC) dosage (5–40 mg/L), contact time (0–240 min), temperature (15–35 °C), and initial pH (2–11)—on the adsorption of MIB and DMDS were systematically investigated. Upon reaching the predetermined adsorption time, the water samples were filtered through a 0.45 μm water-based membrane. The concentrations of MIB and DMDS in the filtrate were quantified by GC-MS (Agilent 7890B GC coupled with 5977B MSD, Agilent Technologies, Santa Clara, CA, USA), and the equilibrium mass concentration was calculated based on the standard curve fitting equation. The equilibrium adsorption capacity (*Q_e_*, ng/mg) and the removal rate (*E*, %) were subsequently calculated using Equations (1) and (2), respectively.

(1) $$Q_{e}=\left(C_{0}-C_{e}\right)\times \frac{V}{m}$$

(2) $$E=\frac{C_{0}-C_{e}}{C_{0}}\times 100\%$$

where *C*_0_ is the concentration value measured in the control group at the end of the experiment, ng/L; *C_e_* is the mass concentration of the solution at the adsorption equilibrium, ng/L; V is the volume of the solution, L; and m is the mass of AC added, mg.

Adsorption kinetics experiments were performed with CSC, CAC, and SAC in 250 mL conical flasks. A consistent PAC dosage of 40 mg/L and an initial MIB/DMDS concentration of 2000 ng/L were applied for all kinetics tests. Samples were collected at predetermined time intervals of 0, 1, 3, 5, 10, 10, 20, 30, 60, 120, 180, and 240 min to monitor the adsorption process over time.

For the adsorption isotherm experiments, CSC, CAC, and SAC were evaluated in 1 L dissolved oxygen bottles. The PAC dosage varied from 0 to 40 mg/L while the initial MIB/DMDS concentration was maintained at 2000 ng/L. After an adsorption period of 240 min, which was determined from the kinetics study to be sufficient for reaching equilibrium, all samples were processed. Each experimental condition was conducted in triplicate to ensure data reliability. The samples were filtered through 0.45 μm membranes, and the concentrations of MIB and DMDS in the filtrate were quantified following the analytical procedure detailed in Section 3.3.1. Definitions and formula explanations for all models can be found in the Supplementary Materials.

### 3.3. Methods of Analysis

#### 3.3.1. Analytical Method for MIB and DMDS Quantification

The concentration of MIB and DMDS in water was determined by headspace solid-phase microextraction (HS-SPME) coupled with gas chromatography–mass spectrometry (GC-MS Agilent 7890B GC coupled with 5977B MSD, Santa Clara, CA, USA) under the following conditions: sample volume, 10 mL; mass of NaCl, 2.5 g; chromatographic column, HP5-MS quartz capillary column (30 m × 0.25 mm, film thickness: 0.25 μm); temperature increase program, 40 °C for 4 min, increased to 250 °C at 10 °C/min and kept for 5 min; inlet temperature, 250 °C; carrier gas (He) flow rate, 0.8 mL /min; ion source temperature, 230 °C, quadrupole temperature, 150 °C; auxiliary heating zone temperature, 280 °C; solvent delay time, 8 min. The standard curves *R*^2^ are greater than 0.995 [51] (Figures S1 and S2; Tables S2 and S3).

#### 3.3.2. Characterization of PAC

The specific surface area and pore volume of PAC were obtained using a fully automated specific surface area and porosity analyzer (BSD-660 A3M, Beishide Instrument Technology (Beijing) Co., Ltd., Beijing, China). The measurements were based on N_2_ adsorption and degassing isotherms, BET-specific surface area is derived from the BET theory, micropore parameters are based on the T-Plot theory, while mesopore/macropore parameters originate from the BJH theory (desorption branch). FTIR absorption spectra (TENSORп, Tensor AG, Genderkingen, Germany) were determined by FTIR spectrometer in the range of 4000–400 cm^−1^ to characterize its surface functional groups. SEM images (7610F, JEOL, Tokyo, Japan) were obtained by a scanning electron microscope to examine the surface morphology of ACs. XRD patterns were recorded using a D8 FOCUS lynxeye detector (Bruker AXS, Karlsruhe, Germany) with Cu-Kα radiation (λ = 1.5406 Å). The data were collected in the 2θ range of 5° to 80° at a scanning speed of 2°/min and a step size of 0.02°.

The specific procedure of Boehm titration was as follows: three 0.5 g portions of each material analyzed were added to 25 mL of NaOH, Na_2_CO_3_, and NaHCO_3_ 0.1 M solution. The suspension was magnetically stirred for 24 h and then filtered to remove solids, and aliquots of 10 mL were taken. Each solution was acidified by adding 20 mL of 0.1 M HCl, with the exception of the Na_2_CO_3_ solution, of which 30 mL was used because it is a dibasic base. The solutions were then back-titrated with 0.1 M NaOH solution, using phenolphthalein (indicator, reagent grade, Sigma Aldrich) as the endpoint indicator. Solutions were prepared using NaOH (>98%, Sigma Aldrich, Saint Louis, MO, USA), NaHCO_3_ (>99.5%, Sigma Aldrich), Na_2_CO_3_ (>99.5%, Sigma Aldrich), HCl (1 M, Fisher Scientific, Waltham, MA, USA), and ultra-high purity 18.2 MΩ cm Milli-Q water. The concentrations of hydroxyl, lactone, and carboxyl groups were then calculated assuming that NaHCO_3_ neutralized only the carboxyl group, Na_2_CO_3_ neutralized the carboxyl group and lactone, and NaOH neutralized the carboxyl, lactone, and hydroxyl groups. The total acid group is calculated as the sum of the hydroxyl, lactone, and carboxyl groups. Quantitative experiments were performed in triplicate, and the error is the standard deviation of three experiments [52].

### 3.4. Reusability Evaluation of CSC

To reusability of CSC was evaluated through a series of desorption and regeneration experiments. After adsorption treatment under optimal conditions, CSC adsorbed with MIB /DMDS was separated. Then, CSC loaded with MIB/DMDS was placed in contact with 100 mL of a 1:9 (*v*/*v*) acetic acid and methanol solution used as eluent. The desorption process was carried out in a Soxhlet extractor for 3 h. After the desorption process, the CSC was thoroughly washed several times with distilled water and then dried in an oven. The adsorption–desorption processes were carried out for a maximum of five cycles to check the efficiency of CSC in removing MIB or DMDS under regeneration conditions [53].

## 4. Conclusions

This study systematically evaluated the adsorption performance of three ACs—CSC, CAC, and SAC—for the removal of typical algal odorants, MIB and DMDS. The initial comparative screening identified CSC as the superior adsorbent, achieving removal efficiencies of 87.41% for MIB and 71.20% for DMDS at a dosage of 20 mg/L, attributable to its well-developed hierarchical pore structure and optimal hydrophobicity. Consequently, CSC was selected as the model adsorbent for subsequent in-depth mechanistic investigations. The adsorption process for both MIB and DMDS was determined to be monolayer coverage on a homogeneous surface, as best described by the Langmuir isotherm model. Kinetic analysis revealed distinct adsorption behaviors: MIB adsorption followed a pseudo-second-order model, while DMDS adsorption was better fitted by a pseudo-first-order model. Thermodynamic studies confirmed that the adsorption onto CSC was spontaneous, endothermic, and physically dominated, with no new chemical bonds formed, as verified by FTIR. Furthermore, CSC demonstrated excellent regeneration potential, retaining 85.89% and 68.49% of its initial adsorption capacity for MIB and DMDS, respectively, after five adsorption–desorption cycles. This study provides a theoretical basis for the screening and optimization of AC materials in the removal of water odorants.

## Figures and Tables

**Table 1:** Characterization of the pore structure of different types of AC.

Materials	BET SSA (m^2^ g^−1^)	V_t_ (cm^3^ g^−1^)	S_meso_ (m^2^ g^−1^)	V_meso_ (cm^3^ g^−1^)	S_micro_ (m^2^ g^−1^)	V_micro_ (cm^3^ g^−1^)	Average Pore Size (nm)
CAC	984.9779	0.5595	337.8621	0.2777	856.3402	0.3710	2.2721
CSC	1264.6279	1.0317	524.6927	0.7019	878.3475	0.3630	3.2633
SAC	299.3159	0.1849	108.5387	0.0983	254.9459	0.1086	2.4710

**Table 2:** Kinetic fitting parameters.

Compounds	Kinetic Model	Parameters	Types of AC		
			CAC	CSC	SAC
MIB	Pseudo-first-order	*qe*/(ng/mg)	40.5437	42.8033	34.0526
		*K*_1_ (min^−1^)	0.05146	0.06003	0.05953
		*R* ^2^	0.9576	0.9823	0.9439
	Pseudo-second-order	*qe*/(ng/mg)	44.9475	47.3695	37.3408
		*K*_2_	0.00151	0.00162	0.00218
		*R* ^2^	0.9867	0.9969	0.9780
	Elovich model	*α*	6.6108	7.7941	7.6184
		*β*	0.1158	0.1109	0.1479
		*R* ^2^	0.9909	0.9788	0.9848
	Weber–Morris	*K*_1_	5.33	6.81	4.12
		*C*_1_	0.24	−3.60	3.24
		*K*_2_	2.26	1.54	1.05
		*C*_2_	17.76	27.41	22.69
		*K*_3_	0.2	0.27	0.02
		*C*_3_	39.45	40.55	32.23
		*R* ^2^	0.9918	0.9880	0.9702
DMDS	Pseudo-first-order	*qe*/(ng/mg)	34.1751	36.0123	29.0173
		*K*_1_ (min^−1^)	0.0198	0.0203	0.0180
		*R* ^2^	0.9927	0.9951	0.9939
	Pseudo-second-order	*qe*/(ng/mg)	44.2102	45.1435	37.9584
		*K*_2_	0.000395	0.000422	0.000417
		*R* ^2^	0.9856	0.9885	0.9879
	Elovich model	*α*	0.8642	1.0245	0.6795
		*β*	0.0750	0.0788	0.0864
		*R* ^2^	0.9760	0.9798	0.9798
	Weber–Morris	*K*_1_	3.67	3.44	2.81
		*C*_1_	−4.96	−3.36	−3.65
		*K*_2_	0.19	0.15	0.06
		*C*_2_	30.07	32.70	26.61
		*R* ^2^	0.9874	0.9915	0.9914

**Table 3:** Isotherm fitting parameters.

Compounds	Isotherm Model	Parameters	Types of AC		
			CAC	CSC	SAC
MIB	Langmuir model	*Q*_0_/(ng/mg)	285.1784	304.1376	280.6320
		*K_L_* (L/mg)	0.00189	0.0022	0.00323
		*R_L_*	0.209	0.185	0.134
		*R* ^2^	0.9282	0.9428	0.9636
	Freundlich model	*K_F_*/(ng^(1−1/n)^L^−1^g^−1^)	7.4989	12.9356	3.0294
		*1/n*	0.4605	0.4392	0.562
		*R* ^2^	0.9729	0.9257	0.9879
	Temkin model	*K_T_* (L/mg)	0.03178	0.0284	0.01201
		*B_T_* (J mol^−1^)	52.1317	60.0906	62.1558
		*R* ^2^	0.9785	0.9145	0.9585
DMDS	Langmuir model	*Q*_0_/(ng/mg)	209.5339	224.7103	275.2114
		*K_L_* (L/mg)	0.0068	0.0114	0.0054
		*R_L_*	0.068	0.079	0.085
		*R* ^2^	0.9864	0.9320	0.9828
	Freundlich model	*K_F_*/(ng^(1−1/n)^L^−1^g^−1^)	0.3648	11.0081	3.5039
		*1/n*	0.3711	0.3711	0.4738
		*R* ^2^	0.9661	0.9084	0.9846
	Temkin model	*K_T_* (L/mg)	0.00338	0.0336	0.0231
		*B_T_* (J mol^−1^)	39.9146	41.8064	31.7653
		*R* ^2^	0.9926	0.9329	0.9728

**Table 4:** Thermodynamic parameters of CSC at different temperatures.

Compounds	*T*/(K)	Δ*H*/(kJ·mol^−1^)	Δ*S*/(J·mol^−1^·K^−1^)	Δ*G*/(kJ·mol^−1^)
MIB	288	8.4928	49.54	−5.77
	298			−6.34
	308			−6.78
DMDS	288	8.8361	49.65	−5.46
	298			−5.94
	308			−6.45

## Data Availability

The authors do not have permission to share data.

## Supplementary Materials

The following supporting information can be downloaded at https://www.mdpi.com/article/10.3390/molecules30224348/s1. Figure S1: Standard curves for MIB and DMDS; Figure S2: Chromatograms of MIB and DMDS; Figure S3: Adsorption isotherm model: (a) 288 K; (b) 298 K; (c) 308 K; Figure S4: Van’t Hoff equation solving ∆H, ∆S, ∆G; Table S1: Basic physical and chemical properties of MIB and DMDS; Table S2: Detection limit and accuracy of the method; Table S3: Retention time of MIB and DMDS, qualitative and quantitative ions; Table S4: Adsorption isotherms of CSC at 288 K, 298 K, 308 K; Table S5: Thermodynamic Calculation Parameters. Refs. [54,55,56,57,58] are cited in the Supplementary Materials.

## Abbreviations

The following abbreviations are used in this manuscript:

PAC	powdered activated carbon
CAC	coal-based activated carbon
CSC	coconut shell-derived activated carbon
SAC	Sargassum activated carbon
MIB	2-methylisoborneol
DMDS	dimethyl disulfide
PFO	pseudo-first-order model
PSO	pseudo-second-order model
