# Supplementary material for: Adsorption of Algal-Derived 2-Methylisoborneol (MIB) and Dimethyl Disulfide (DMDS) onto Activated Carbon: The Role of Pore Structure and Hydrophobicity
Source: Molecules. 2025 Nov 10;30(22):4348. doi: 10.3390/molecules30224348 (PMC12654551; doi:10.3390/molecules30224348)
Supplement: Supplementary file 1 [file molecules-30-04348-s001.zip › molecules-3961279-supplementary.pdf]

Supplementary Materials

Adsorption of 2-methylisocaprolactone (MIB) and dimethyl disulfide (DMDS) onto Powered Activated Carbon: A Comparative Analysis of Key factors, Kinetics, Mechanisms and Regeneration Performance

Yuqin Zhao [1], Yulan Zhao [1], Hui Guo [1], Denghui Peng [2,3,*], Wenwen Kong [2,3,*], Fengjian Yan [1,†], Shumei Zhou [1,†], Quansheng Li [1,†], Boxiong Shen [2,3,*] and Chongrui Lyu [2,3]

1 Tianjin Water Group Limited Company, Yinluan Erwangzhuang Branch, Tianjin 301802, China

2 Hebei Engineering Research Center of Pollution Control in Power System, School of Energy and Environmental Engineering, Hebei University of Technology, Tianjin 300401, China

3 Tianjin Key Laboratory of Clean Energy and Pollution Control, School of Energy and Environmental Engineering, Hebei University of Technology, Tianjin 300401, China

* Correspondence: pdh1043692470@163.com (D.P.); 2023036@hebut.edu.cn (W.K.); shenbx@hebut.edu.cn (B.S.); Tel.: +86-17814776318 (D.P.); +86-15822056950 (W.K.); +86-18622132754 (B.S.)

† These authors contributed equally to this work.

Contents

Table S1. Basic physical and chemical properties of MIB and DMDS

Table S2. Detection limit and accuracy of the method

Table S3. Retention time of MIB and DMDS, qualitative and quantitative ions

Table S4. Adsorption isotherms of CSC at 288K,298K,308K

Table. S5 Thermodynamic calculation parameters

Figure S1 Standard curves for MIB and DMDS

Figure S2 Chromatograms of MIB and DMDS

Figure S3 Adsorption isotherm model: (a) 288K; (b) 298 K; (c) 308K

Figure S4 Van't Hoff equation solving ΔH, ΔS, ΔG

To investigate the adsorption kinetics of ACs on MIB and DMDS, the experimental results were fitted using the pseudo-first-order model (PFO) (Equation (S3)) [1], pseudo-second-order model (PSO) (Equation (S4)) [1], Elovich model (Equation (S5)) [2], and Weber-Morris model, respectively.

$$ln(Q_e - Q_t) = lnQ_e - K_1 t \quad \text{(S3)}$$

$$\frac{t}{Q_t} = \frac{1}{K_2 Q_e^2} + \frac{t}{Q_e} \quad \text{(S4)}$$

$$Q_t = \frac{1}{\beta ln(\alpha\beta t + 1)} \quad \text{(S5)}$$

$$Q_t = \text{Kt\textasciicircum 1/2 + C} \quad \text{(S6)}$$

where Qt and Qe are the adsorption capacity at the moment t and after adsorption equilibrium, ng/mg, respectively; K_1 and K_2 are the adsorption rate constants for quasi-primary and quasi-secondary kinetics, respectively; α is the initial adsorption rate; β is the adsorption constant; K is the internal diffusion rate constant; and C is a constant related to the boundary layer.

The adsorption isotherm is a curve that reflects the relationship between the concentration of adsorbate molecules in the original solution and on the surface of the adsorbent when the adsorption and removal processes between the original solution and the surface of the adsorbent reach equilibrium at a certain temperature. In this study, three kinds of more widely used isotherm models are considered, as follows:

Langmuir model [3]:

$$Q_e = \frac{Q_0 K_L C_e}{1 + K_L C_e} \quad \text{(S7)}$$

$$R_L = \frac{1}{1 + K_L C_0} \quad \text{(S8)}$$

Freundlich model [4]:

$$Q_e = K_F C_e^{\frac{1}{n}} \quad \text{(S9)}$$

Temkin model [5]:

$$Q_e = B_T ln\,(K_T C_e) \quad \text{(S10)}$$

where C_e is the residual concentration of odorant in solution at adsorption equilibrium (ng/L); C_0 is the initial concentration of odor (ng/L); Q_0 (ng/mg) and K_L (L/mg) are parameters of the Langmuir isothermal adsorption model; K_F ($ng^{(1-1/n)}$ $L^{(1/n)}$ g^{-1}) and 1/n are Freundlich isothermal adsorption model parameters; and K_T (L/mg) and B_T(J mol^{-1}) are Temkin isothermal adsorption model parameters.

Table S1. Basic physical and chemical properties of MIB and DMDS

The analytical method was verified from the aspects of linearity, the limit of detection, the limit of quantification, precision, and accuracy. Six standard solutions of different

T&O Compounds	Chemical Formula	Basic Structural Unit	OTC (ng/L)	Solubility (mg/cm^3)	RMS (g/mol)
MIB	$C_{11}H_{20}O$	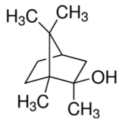	10	0.45	168.276
DMDS	$C_2H_6S_2$	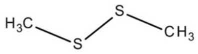	30	insoluble in water	94.199

analytes with increasing concentrations were prepared. Under optimized conditions, a standard curve was established using selective ion scanning (SIM) with the peak area (y) of the substance to be quantified as the ordinate and the mass

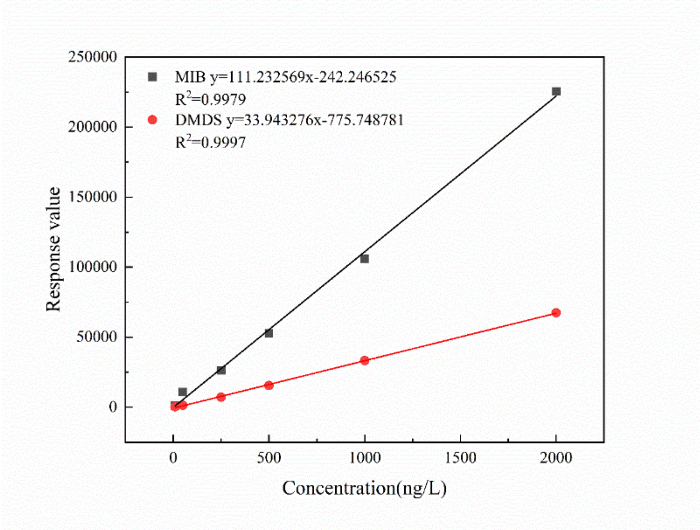

concentration (x) of the substance to be quantified as the abscissa, and the slope of the correction curve was determined to estimate the sensitivity.

Figure S1 Standard curves for MIB and DMDS

The concentration in the sample was determined by six parallel determinations following the sample analysis steps, and the concentration in the sample was measured by the formula MDL=t(n-1,0.99)×S, where MDL is the method detection limit, n is the number of parallel determinations of the sample, t is the t-distribution (one-sided) with a degree of freedom of n-1 and a confidence interval of 99%, and S is the standard deviation of the nth parallel determination, when n=6 and t=3.365.

Table S2. Detection limit and accuracy of the method

Compounds	Concentration (ng/L)	Results of 6 parallel tests						Standard deviation (ng/L)	Detection limit (ng/L)	Accuracy /%
		1	2	3	4	5	6			
MIB	50	55.1799	53.9836	57.2471	56.9655	55.8010	55.2146	1.114	3.7486	2.00
DMDS		55.7456	57.3024	53.1157	57.9465	55.2367	57.9803	1.736	5.8416	3.09

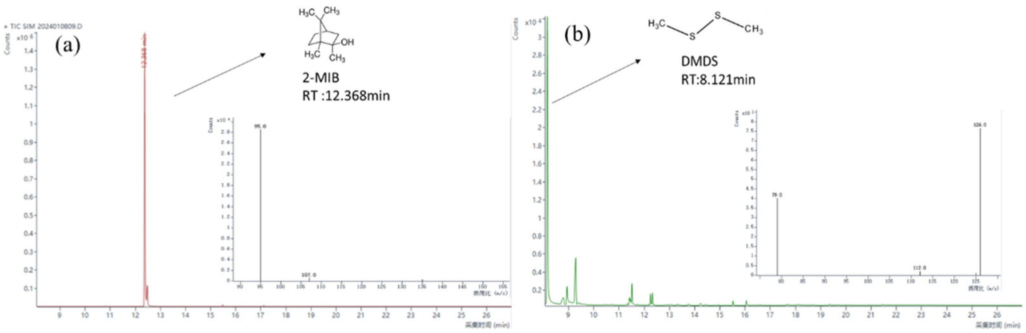

Figure S2 Chromatograms of MIB and DMDS

Table S3. Retention time of MIB and DMDS, qualitative and quantitative ions

Compounds	Retention time (min)	Mass-to-charge ratio(m/z)	
		Characterized ion	Quantitative ion
MIB	12.368	95 107 135	95
DMDS	8.121	79 112 126	126

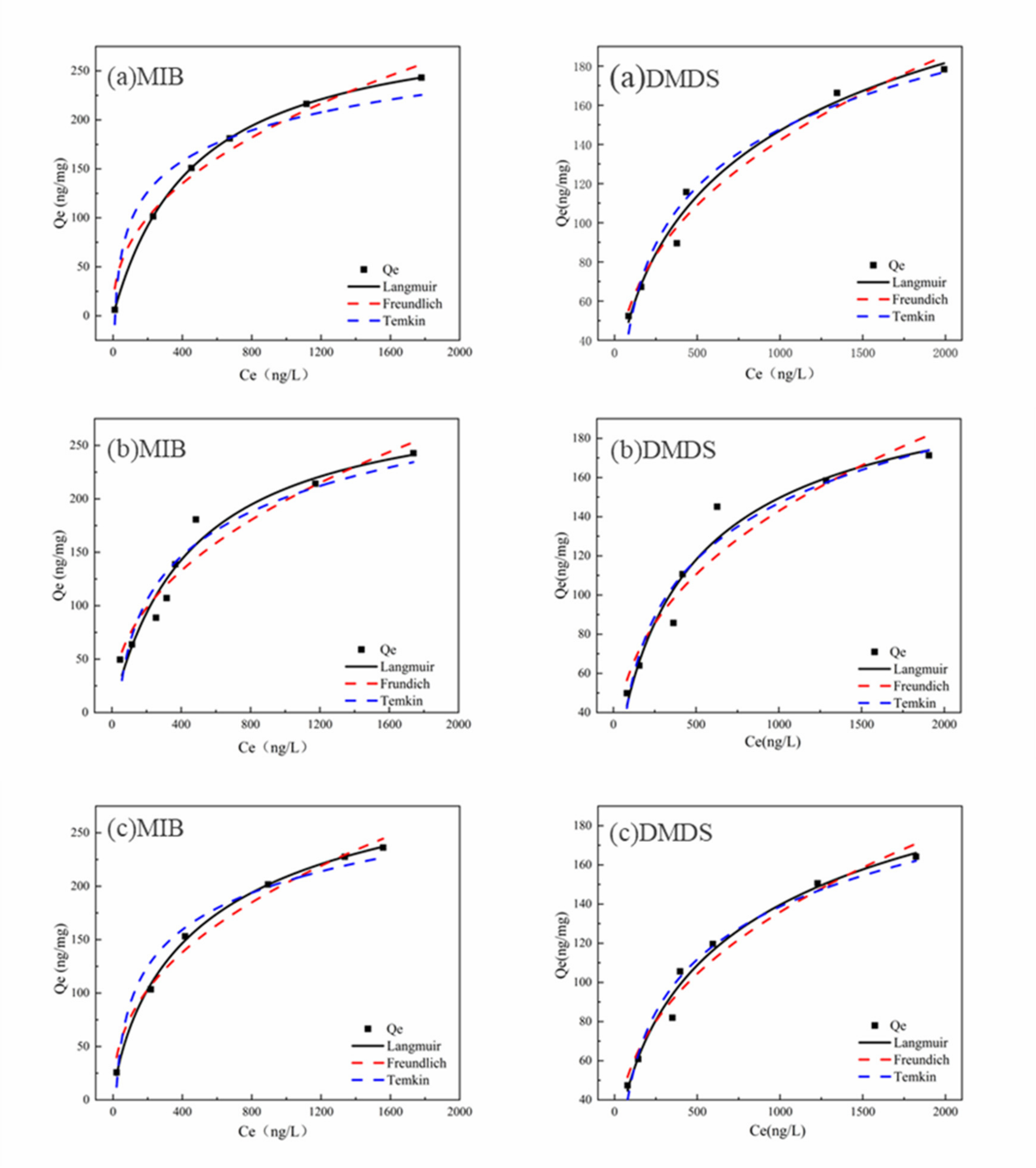

Figure S3 Adsorption isotherm model：(a)288K；(b) 298 K；(c) 308K

Table S4. Adsorption isotherms of CSC at 288K,298K,308K

Compounds	Isotherm model	Parameters	Temperature		
			288K	298 K	308K
MIB	Langmuir model	Q_0/(ng/mg)	350.6483	304.1376	306.6202
		K_L(L/mg)	0.0066	0.0022	0.0021
		R_L	0.070	0.185	0.192
		R^2	0.9978	0.9428	0.9998
	Freundlich model	K_F/(ng$^{(1-1/n)}$L $^{-1}$g $^{-1}$)	11.1554	12.9356	14.1455
		1/n	0.4200	0.4392	0.4299
		R^2	0.9809	0.9257	0.9685
	Temkin model	$K_T(L/mg)$	0.0616	0.0284	0.0818
		$B_T(J\ mol^{-1})$	49.7087	60.0906	45.2891
		R^2	0.9602	0.9145	0.9381

DMDS	Langmuir model	Q_0/(ng/mg)	273.3855	224.7103	321.2884
		K_L(L/mg)	0.0110	0.0114	0.0058
		R_L	0.043	0.079	0.042
		R^2	0.9728	0.9320	0.9698
	Freundlich model	K_F/(ng$^{(1-1/n)}$L^{-1}g^{-1})	9.79414	11.0081	12.5023
		1/n	0.3807	0.3711	0.3832
		R^2	0.9647	0.9084	0.9685
	Temkin model	$K_T(L/mg)$	0.0353	0.0336	0.0328
		$B_T(J\ mol^{-1})$	38.9289	41.8064	42.3067
		R^2	0.9644	0.9329	0.9605

Based on the basic concepts of thermodynamics, assuming that energy cannot be gained or lost in a stand-alone system, the entropy change is the only driving force for the reaction. However, in practical applications, both the energy factor ΔH and the entropy factor ΔS must be considered to determine which process will be spontaneous. It is common to determine whether a reaction is spontaneous or not by the Gibbs free energy ΔG, which can be determined by the classical Van't Hoff equation [4]:

$$\Delta G = -RTLnK_{ad} \tag{S11}$$

where R is the universal gas constant (8.314 × 10^{-3} KJmol^{-1}K^{-1}); T is the absolute temperature in K; and K_{ad} is the adsorption equilibrium constant, which can be equated by the equilibrium constant K_F when the adsorbate is weakly polarized [5]. In turn, ΔG can be determined by the following equation:

$$\Delta G = \Delta H - T\Delta S \tag{S12}$$

Combining the equations gives

$$lnK_{ad} = \Delta SR^{-1} - \Delta HR^{-1}T^{-1} \tag{S13}$$

where Kad at three temperatures (288K, 298K, 308K) is the dimensionless constant obtained by taking ln(Q*e*/Ce) as the

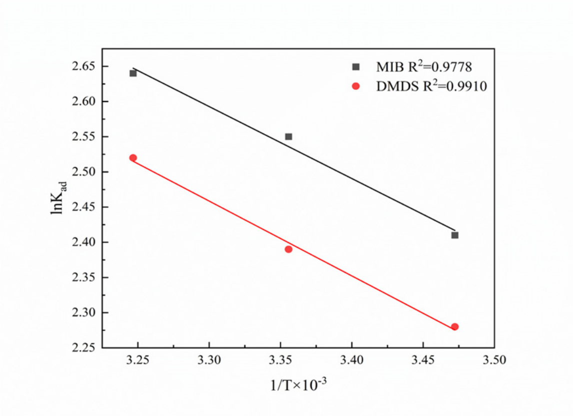

vertical coordinate and Q*e* as the horizontal coordinate to determine a straight line with Q*e* =0. Substituting this into the equation gives Figure S5.

Figure S4 Van't Hoff equation solving Δ*H*, Δ*S*, Δ*G*

Table S5 Thermodynamic calculation parameters

T	Kad	1/T*10^{-3}	lnKad	intercept(ΔS/R)	Slope(-ΔH/R)	R^2	R(J/mol*K)	ΔG=-RTlnKad(**kJ·mol^{-1}**)	ΔH(**kJ·mol^{-1}**)	ΔS(**J·mol^{-1}·K^{-1}**)
288	11.1554	3.47	2.4119	5.9653	-1.0215	0.9778	8.314	-5.7752	8.4928	49.5418
298	12.9356	3.36	2.5600					-6.3425		
308	14.1455	3.25	2.6494					-6.7843		
288	9.79414	3.47	2.2818	5.9659	-1.0628	0.9910		-5.4636	8.8361	49.6517
298	11.0081	3.36	2.3986					-5.9428		
308	12.5023	3.25	2.5259					-6.4681		

1. Azizian, S. Kinetic models of sorption: A theoretical analysis. *J. Colloid Interface Sci.* **2004**, *276*, 47–52. https://doi.org/10.1016/j.jcis.2004.03.048.
2. Zango, Z.U.; Bakar, N.H.H.A.; Sambudi, N.S.; Jumbri, K.; Abdullah, N.A.F.; Kadir, E.A.; Saad, B. Adsorption of chrysene in aqueous solution onto MIL-88(Fe) and NH2-MIL-88(Fe) metal-organic frameworks: Kinetics, isotherms, thermodynamics and docking simulation studies. *J. Environ. Chem. Eng.* **2020**, *8*, 103544. https://doi.org/10.1016/j.jece.2019.103544.
3. Ajmani, A.; Shahnaz, T.; Subbiah, S.; Narayanasamy, S. Hexavalent chromium adsorption on virgin, biochar, and chemically modified carbons prepared from Phanera vahlii fruit biomass: Equilibrium, kinetics, and thermodynamics approach. *Environ. Sci. Pollut. Res.* **2019**, *26*, 32137–32150. https://doi.org/10.1007/s11356-019-06335-z.
4. Agarwal, A.; Kumar, A.; Gupta, P.; Tomar, R.; Singh, N.B. Cu (II) ions removal from water by charcoal obtained from marigold flower waste. *Mater. Today Proc.* **2019**, *34*, 875–879. https://doi.org/10.1016/j.matpr.2020.11.046.
5. Lima, E.C.; Hosseini-Bandegharaei, A.; Moreno-Piraján, J.C.; Anastopoulos, I. A critical review of the estimation of the thermodynamic parameters on adsorption equilibria. Wrong use of equilibrium constant in the Van't Hoof equation for calculation of thermodynamic parameters of adsorption. *J. Mol. Liq.* **2018**, *273*, 425–434. https://doi.org/10.1016/j.molliq.2018.10.048.

Disclaimer/Publisher's Note: The statements, opinions and data contained in all publications are solely those of the individual author(s) and contributor(s) and not of MDPI and/or the editor(s). MDPI and/or the editor(s) disclaim responsibility for any injury to people or property resulting from any ideas, methods, instructions or products referred to in the content.
